# NF-κB1 inhibits c-Myc protein degradation through suppression of FBW7 expression

**DOI:** 10.18632/oncotarget.1643

**Published:** 2014-01-15

**Authors:** Haishan Huang, Li Ma, Jingxia Li, Yonghui Yu, Dongyun Zhang, Jinlong Wei, Honglei Jin, Derek Xu, Jimin Gao, Chuanshu Huang

**Affiliations:** ^1^ Zhejiang Provincial Key Laboratory for Technology & Application of Model Organisms, School of Life Sciences, Wenzhou Medical University, Wenzhou, Zhejiang, China; ^2^ Nelson Institute of Environmental Medicine, New York University School of Medicine, Tuxedo, NY, USA; ^3^ Jericho High School, Jericho, NY

**Keywords:** NF-κB p50, arsenite, c-Myc, protein degradation, FBW7

## Abstract

NF-κB is a well-known transcription factor in regulation of multiple gene transcription and biological processes, and most of them are relied on its transcriptional activity of the p65/RelA subunit, while biological function of another ubiquitously expressed subunit NF-κB1 (p50) remains largely unknown due to lack transcriptional activation domain. Here we discovered a novel biological function of p50 as a regulator of oncogenic c-Myc protein degradation upon arsenite treatment in a NF-κB transcriptional-independent mechanism. Our results found that p50 was crucial for c-Myc protein induction following arsenite treatment by using specific knockdown and deletion of p50 in its normal expressed cells as well as reconstituting expression of p50 in its deficient cells. Subsequently we showed that p50 upregulated c-Myc protein expression mainly through inhibiting its degradation. We also identified that p50 exhibited this novel property by suppression of FBW7 expression. FBW7 was profoundly upregulated in p50-defecient cells in comparison to that in p50 intact cells, whereas knockdown of FBW7 in p50-/- cells restored arsenite-induced c-Myc protein accumulation, assuring that FBW7 up-regulation was responsible for defect of c-Myc protein expression in p50-/- cells. In addition, we discovered that p50 suppressed fbw7 gene transcription via inhibiting transcription factor E2F1 transactivation. Collectively, our studies demonstrated a novel function of p50 as a regulator of c-Myc protein degradation, contributing to our notion that p50-regulated protein expression through multiple levels at protein translation and degradation, further providing a significant insight into the understanding of biomedical significance of p50 protein.

## INTRODUCTION

NF-κB has long been well-documented as a pivotal factor for regulating physiological and pathological processes, including infammation [1], cell survival and anti-apoptosis [2], tumorigenesis [3], immune response and nervous system development [4, 5], all of which largely rely on its transcriptional activity and regulation of its target gene expression. NF-κB is consisted of five distinct members of the Rel family, including NF-κB1 (p50), NF-κB2 (p52), p65 (Rel A), c-Rel and Rel B, forming either homo- or hetero-dimer variants of NF-κB [6]. Of those dimers of NF-κB, p50/p65 is a major one that is predominantly presented and regulated the transcription of its target genes in mammalian cells. In contrast to p65, p50 subunit's contribution to the aforementioned regulations are largely unknown due to lacking a transcriptional domain and therefore unable to act as a transcription factor independently [7], although it has been shown that the p50 homodimer can translocate into the nucleus and bind to NF-κB binding sites of its target genes [8]. Our most recent studies demonstrate that p50 upregulates GADD45α protein expression by promoting its deubiquitination and therefore inhibiting its degradation [9] as well as increases p53 protein translation via modulating the miR190/PHLPPl/Akt-S6 axis [10]. In the light of these findings, we anticipate that p50 is a multi-functional protein that modulates the protein expression at multiple post-transcriptional levels.

Arsenite has been depicted to influence the integrity of mammalian cells, and is a well-documented category of human carcinogen [11], or in some cases as a therapeutic regimen for diseases, including cancers [12]. Our previous studies have demonstrated that arsenite exposure can impact cell transformation [11, 13], whereas arsenite treatment also induces apoptotic responses via a p50-dependent and p65-independent manner [14, 15]. It is important to note that c-Myc has been reported to be an essential component in arsenic-mediated carcinogenesis [16, 17], while c-Myc also acts as a pro-apoptotic protein regulating p53-dependenet or -independent apoptosis [18, 19]. Thus, current study investigated the potential contribution and molecular mechanisms of c-Myc expression to p50-mediated biological effect following arsenite exposure. Here, we discovered that p50 was crucial for c-Myc protein induction via inhibiting its protein degradation rather than via enhancing mRNA transcription. Moreover, we found that FBW7, a tumor suppressor [20], has been identified as a p50 downstream mediator responsible for p50-exerted a novel function on inhibition of c-Myc protein degradation.

## RESULTS

### p50 was required for arsenite-induced c-Myc expression

**Fig 1:**
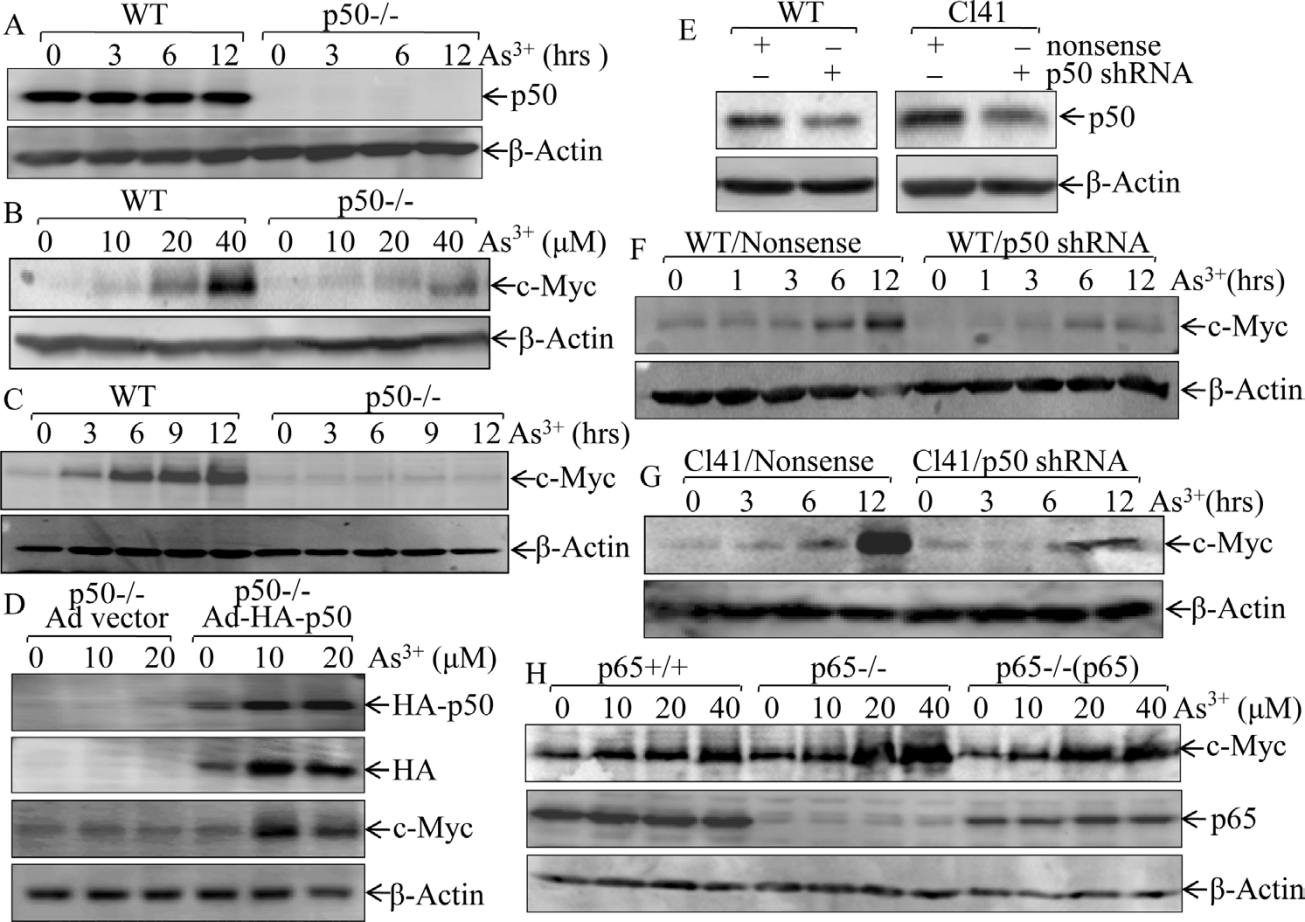
p50, but not p65, upregulates c-Myc expression following arsenite exposure (A), Identification of WT and p50-/-cells; (B-C), WT and p50-/- cells were exposed to indicated concentration of arsenite for 12 h (B), or were treated with 20 μM arsenite for the times indicated (C); (D), p50-/-(Ad vector) and p50-/-(Ad-HA-p50) cells were exposed to indicated doses of arsenite for 12 h; (E-G), shRNA p50 was stably transfected into WT cells and mouse epidermal C141 cells (E), those p50 knockdown transfectants were treated with 20 μM arsenite for different time points as indicated; (H), p65+/+, p65-/-, and p65-/-(p65) cells were treated with arsenite at indicated doses for 12 h. Whole cell extracts from each of above experiments were subjected to Western Blotting for the determination of protein expression of p50, c-Myc, p65, and HA. β-Actin was used as protein loading control.

Human exposure to arsenite is across the lifetime, leading to accumulation of arsenite in tissues [21–24]. Our most recent studies demonstrated that acute exposure to 20μM arsenite shows comparable responses with chronic exposure to 1μM arsenite for two months [25]. Thus, arsenite dose of 20 μM was selected for current short term exposure. Arsenite-induced c-Myc expression has been established for years [26], and NF-κB activation has also been reported to be involved in this process at transcription level [27]. However, there are no report assessing the differential effects of NF-κB p50 and p65 in arsenite-triggered c-Myc expression. To address this issue, mouse embryonic fibroblasts (MEFs) derived from wild-type (WT) or p50 gene knockout (p50-/-) mice were exploited as shown in Fig. 1A, and their responses to arsenite were compared. As shown in Figs. 1B and 1C, depletion of p50 impaired arsenite's impact on c-Myc protein expression, suggesting that p50 promoted c-Myc protein expression in the presence of arsenite. Consistently, reconstitutional expression of p50 in p50-/- cells significantly restored c-Myc induction due to arsenite treatment (Fig. 1D), while knockdown of p50 by specific p50 shRNA in WT MEF cells abolished the c-Myc induction following arsenite treatment (Figs. 1E & 1F). Since skin is one of major targets of arsenite, the mouse epidermal cell line C141 was used to extend our finding that p50 was crucial for c-Myc protein induction due to arsenite treatment (Figs. 1E & 1G). Next we determined the role of p65 in arsenite-induced c-Myc expression, arsenite-induced c-Myc up-regulation slightly increased in p65-/- cells, and was similar in p65-/-(p65). Therefore the aforementioned results demonstrated that arsenite-induced c-Myc expression is predominantly through p50-dependent, and p65-independent manner.

### Arsenite-p50 inhibits c-Myc protein degradation

**Fig 2:**
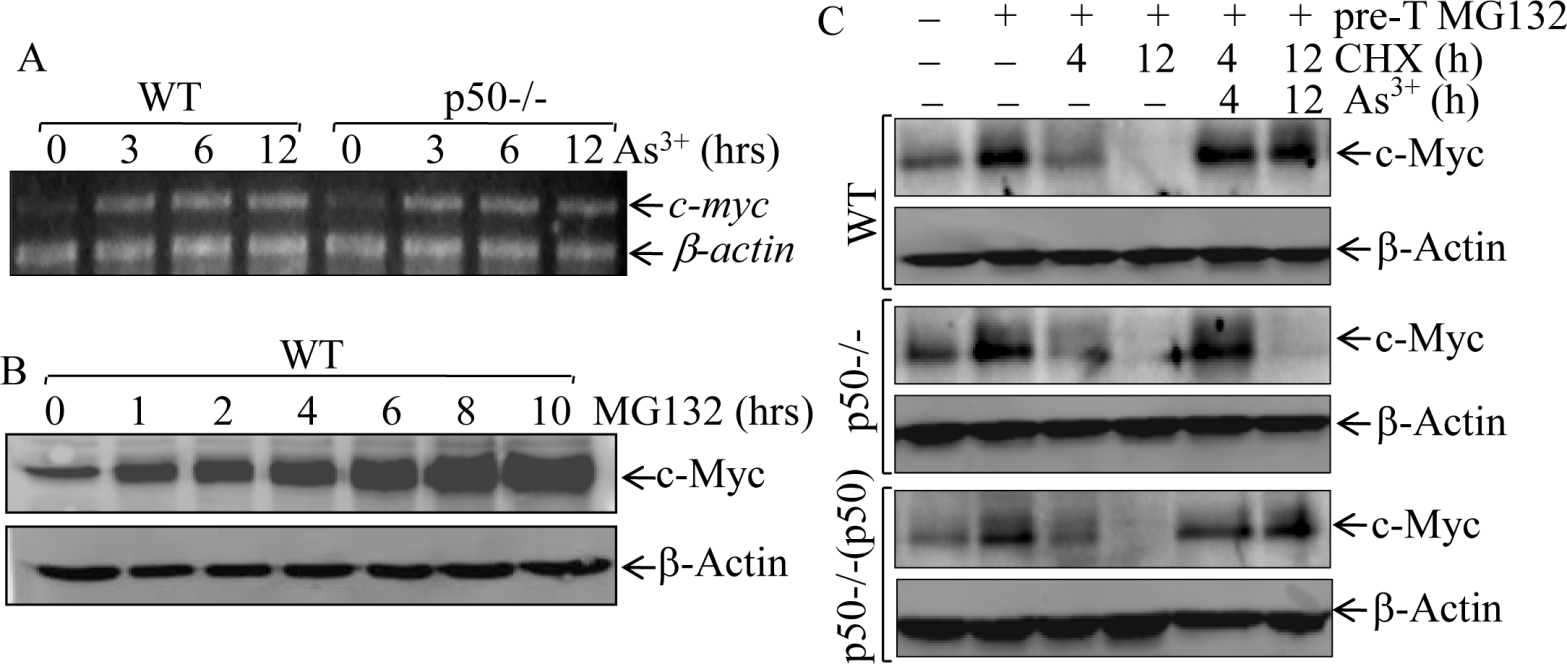
p50 positively regulated c-Myc protein expression through inhibiting its protein degradation (A), WT and p50-/-cells were exposed to 20 μM arsenite and the c-myc mRNA expression was determined by RT-PCR assay; (B), WT cells were pretreated with 10 (μM of MG132 for indicated periods, and the cell extracts were subjected to Western Blotting; (C), WT, p50-/- and p50-/-(p50) cells were pretreated with 10 μM of MG132 for 4h and then exposed to 50 μg/ml CHX in the absence or presence of 20 (μM arsenite for the times indicated after removal of MG132, and the cell extracts were subjected to Western Blotting.

Previous studies have demonstrated that c-Myc protein expression is mainly regulated at levels of mRNA transcription [28] and protein degradation [29]. To elucidate molecular mechanism underlying p50-mediated c-Myc induction by arsenite, we carried out RT-PCR assay to test whether p50 regulates c-Myc mRNA expression, and the results excluded this possibility because there was no observable difference of mRNA induction by arsenite between WT and p50-/- cells (Fig. 2A). We next evaluate potential role of p50 in regulation of c-Myc protein degradation following arsenite exposure. The results obtained from the observation of arsenite effect on the dynamic degradation showed that the pre-accumulated c-Myc proteins gradually (Fig. 2B) disappeared in all three types of cells within 4-12 h after removing of MG132 in the absence of arsenite (Fig. 2C). When arsenite is present in the experimental system, c-Myc protein keeps stable until 12 h after MG132 withdrawal in WT cells, but not in p50-/-cells (Fig. 2C). Notably, re-introduction of p50 into p50-/- cells restored the effect of arsenite on preventing c-Myc protein degradation (Fig. 2C). Based on these results, we conclude that the p50 exerts its effect for up-regulating c-Myc protein expression via inhibiting c-Myc protein degradation following arsenite treatment.

**Fig 3:**
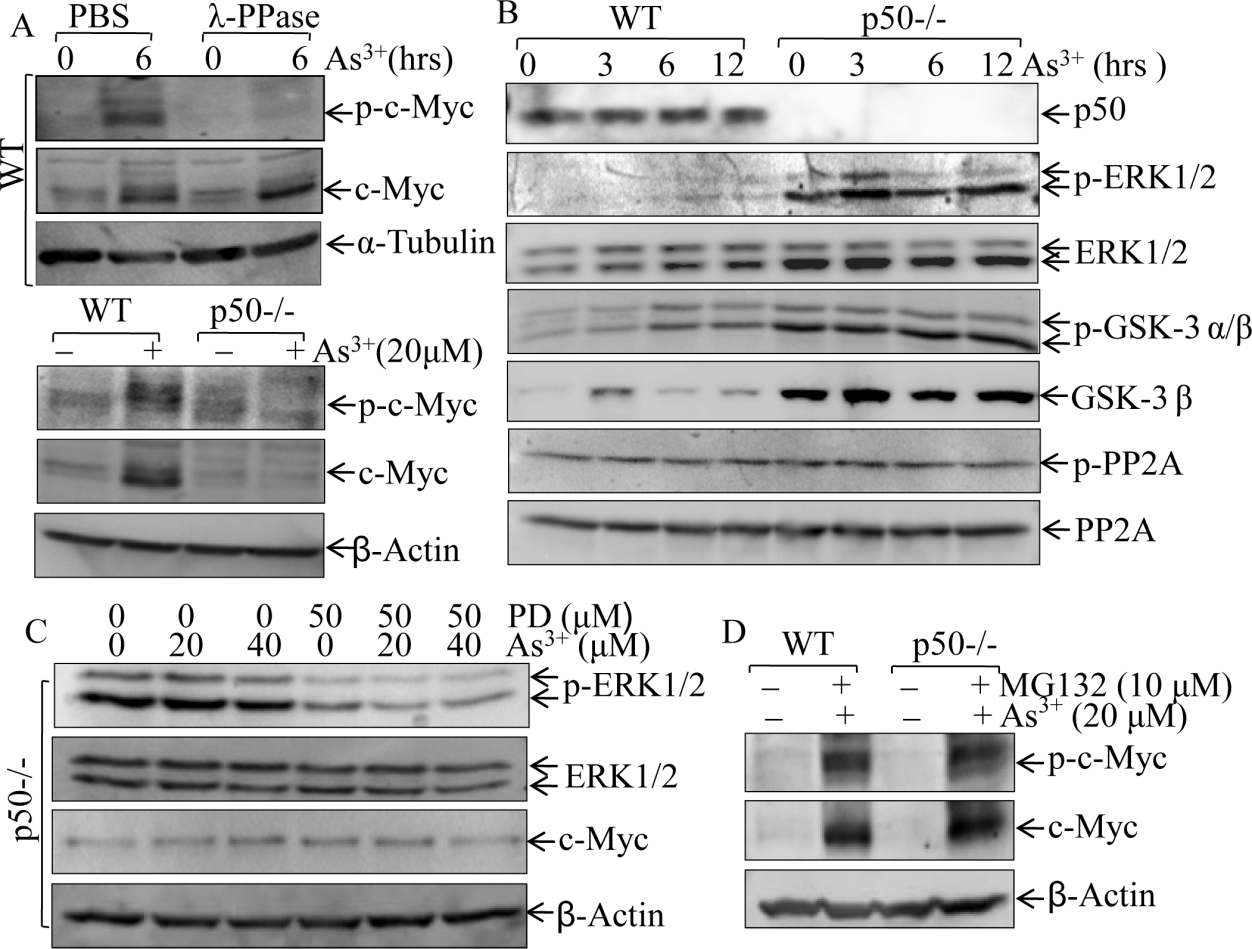
p50 enhanced c-Myc expression through a phosphorylation-independent pathway (A), WT and p50-/- cells were exposed to 20 μM arsenite, and 100μg of whole cell lysate was incubated with λ phosphatase in the phosphatase buffer for 1 h at 37 °C, and phosphorylated status of c-Myc (p-c-Myc) was then analyzed by Western Blotting; (B), WT and p50-/- cells were treated with 20 μM arsenite and cell lysates were subjected to Western Blotting for determination of the p-ERKl/2, ERK1/2, p-GSK3, GSK3, p-PP2A and PP2A; (C), After pre-treatment of cells with MEK1/2 inhibitor PD98059 (50 μM) for 60 min, cells were exposed to arsenite at the indicated doses for 6 h. Cell extracts were subjected to Western Blotting for the determination of p-ERKl/2, ERK1/2 and c-Myc expression; (D), Both WT and p50-/- cells were co-incubated with 10 μM MG132 and 20 μM arsenite for 12 h, and the cell extracts were subjected to Western Blotting.

Phosphorylation is one of important mechanisms for c-Myc protein turnover, which allows the E3 ligase to mediate ubiquitination and degradation of c-Myc protein [30]. Our results indicated that arsenite treatment led to induction of c-Myc protein expression and increased the phosphorylated c-Myc protein, while incubation of this whole cell extracts with λ pptase completely abolished the phosphorylated c-Myc protein, suggesting that arsenite induced accumulation of c-Myc protein expression with induction of c-Myc protein phosphorylation (top panel of Fig. 3A). However, p-c-Myc protein and total c-Myc protein are consistently low in p50-/- cells as compared to those in WT cells (bottom panel of Fig. 3A). We further evaluated the possibility of p50-regulated c-Myc protein phosphorylation by evaluating potential upstream components, such as ERK1/2, GSK3 and PP2A. As shown in Fig. 3B, only ERK1/2 activation was upregulated in p50-/- cells in compared to that in WT cells, which is consistent with down-regulation of c-Myc protein expression in p50-/- cells following arsenite exposure. We, therefore, tested whether inhibition of Erks could rescue c-Myc protein induction by arsenite treatment in p50-/- cells. The results showed that the inhibition of ERK1/2 activation by PD98059 did not substantiate c-Myc protein expression following arsenite treatment (Fig. 3C), suggesting that ERK is not involved in p50-regulated c-Myc protein expression. It has been well characterized that GSK3β phosphorylation negatively regulates its kinase activity and increase c-Myc protein stability [31]. The deletion of p50 led to up-regulation of GSK3β phosphorylation (Fig. 3B) would not support the involvement of GSK3β in p50-mediated upregulation of c-Myc protein expression following arsenite treatment,. Furthermore, accumulated total and phosphorylated c-Myc protein levels by co-treatment of cells with arsenite and MG132 were comparable between WT and p50-/- cells (Fig. 3D). Taken together with our results of no alteration of phosphorylation and expression of PP2A (Fig. 3B), c-Myc protein phosphorylation might not be involved in p50-mediated c-Myc protein expression due to arsenite treatment.

### FBW7 mediates arsenite-p50-inhibited c-Myc degradation

**Fig 4:**
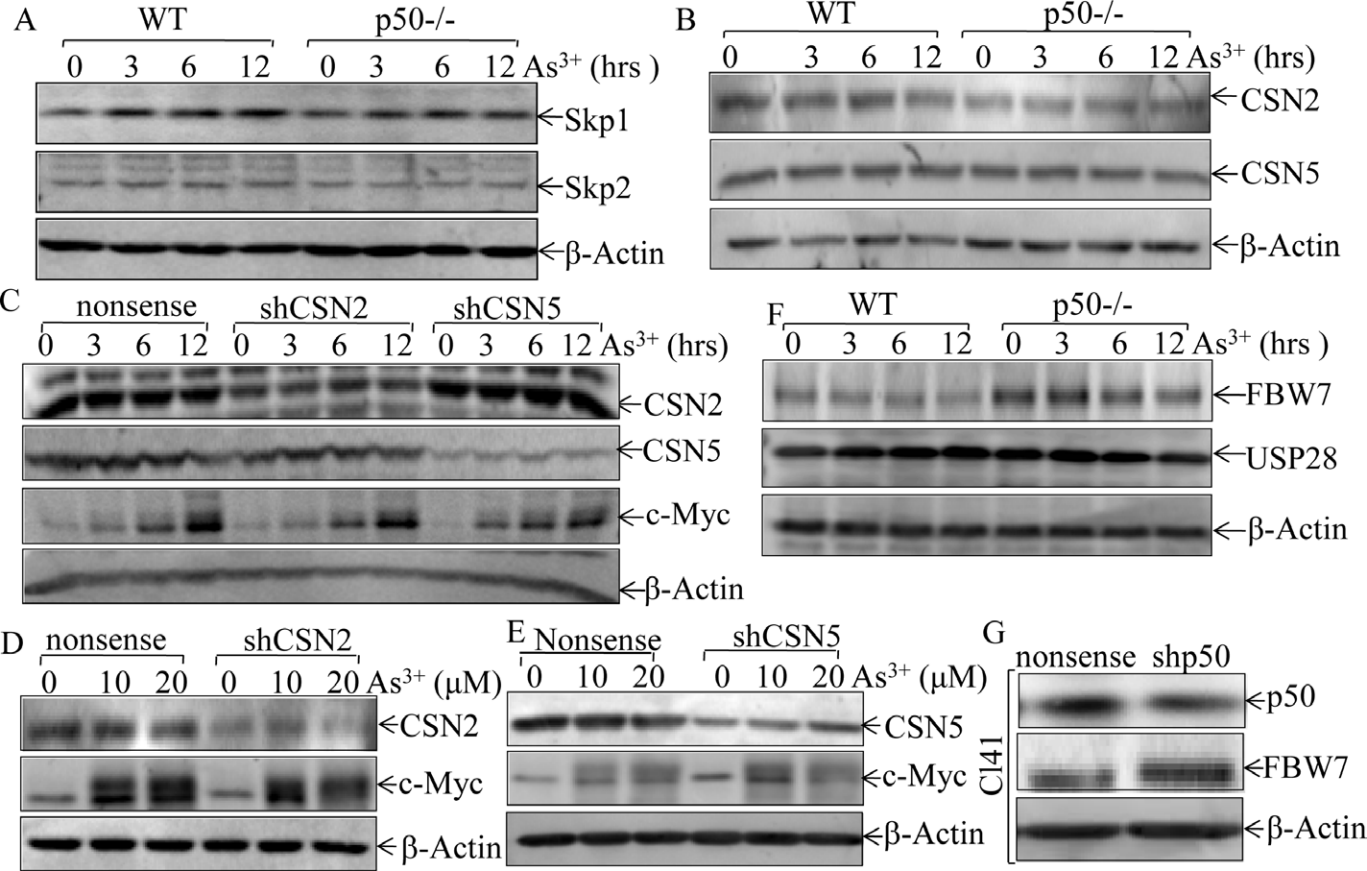
p50 inhibited FBW7 protein expression (A, B, & F), WT and p50-/- cells were exposed to 20 μM arsenite for indicated periods, and cell extracts were subjected to Western Blotting for determination of the expression of Skp1, Skp2, CSN2, CSN5, USP28 and FBW7; (C-E), NIH3T3 cells were stably expressed shCSN2shCSN5 and nonsense vectorand the stable transfectants were exposed to 20 μM arsenite for the indicated periods (C), or treated with arsenite at the indicated concentrations for 12 h (D & E). The cell extracts were subjected to Western Blotting. (G), the cell extracts obtained from Cl41 (shp50) and Cl41 (nonsense) were subjected to Western Blotting for determination of p50 and FBW7 expression.

E3 ligase-mediated protein ubiquitination and degradation is one of the most common mechanisms responsible for protein degradation via a proteasome-dependent manner [32]. Our above results failed to detect the difference of phospho-status of c-Myc between WT and p50-/- cells. With logical following, we next searched for the possible E3 ligase component that might mediate p50-regulated c-Myc protein accumulation following arsenite treatment. Since SKP1, SKP2, USP28, FBW7 and COP9 signalosome (CSN) subunits have been reported to regulate protein degradation [33, 34], we compared those proteins expression between WT and p50-/- cells. As shown in Figs. 4A and 4B, there was no observable difference of protein expression of SKP1, SKP2, CSN2 and CSN5 between WT and p50-/- cells. Moreover, knockdown of CSN2 or CSN5 did not upregulate c-Myc protein expression in NIH3T3 cells (Figs. 4C–4E), excluding the involvement of CSN2 and CSN5 in p50 regulation of c-Myc protein expression upon arsenite treatment. We next compared the protein expression of USP28 and FBW7 between WT and p50-/- cells. As results, FBW7 protein expression was profoundly upregulated in p50-/- cells in comparison to that in WT cells although arsenite treatment only showed a slightly similar downregulation in both types of cells (Fig. 4F). In contrast, the expression of USP28, a key enzyme responsible for deubiquitination of c-Myc, did not show a significant alteration following arsenite treatment between WT and p50-/- cells (Fig. 4F). Moreover, p50-inhibited effect on FBW7 expression was further extended in C141 cells with p50 stable knockdown transfectant (Fig. 4G). Our results strongly indicated that p50 downregulates FBW7 expression and thereby accumulating c-Myc protein following arsenite treatment.

**Fig 5:**
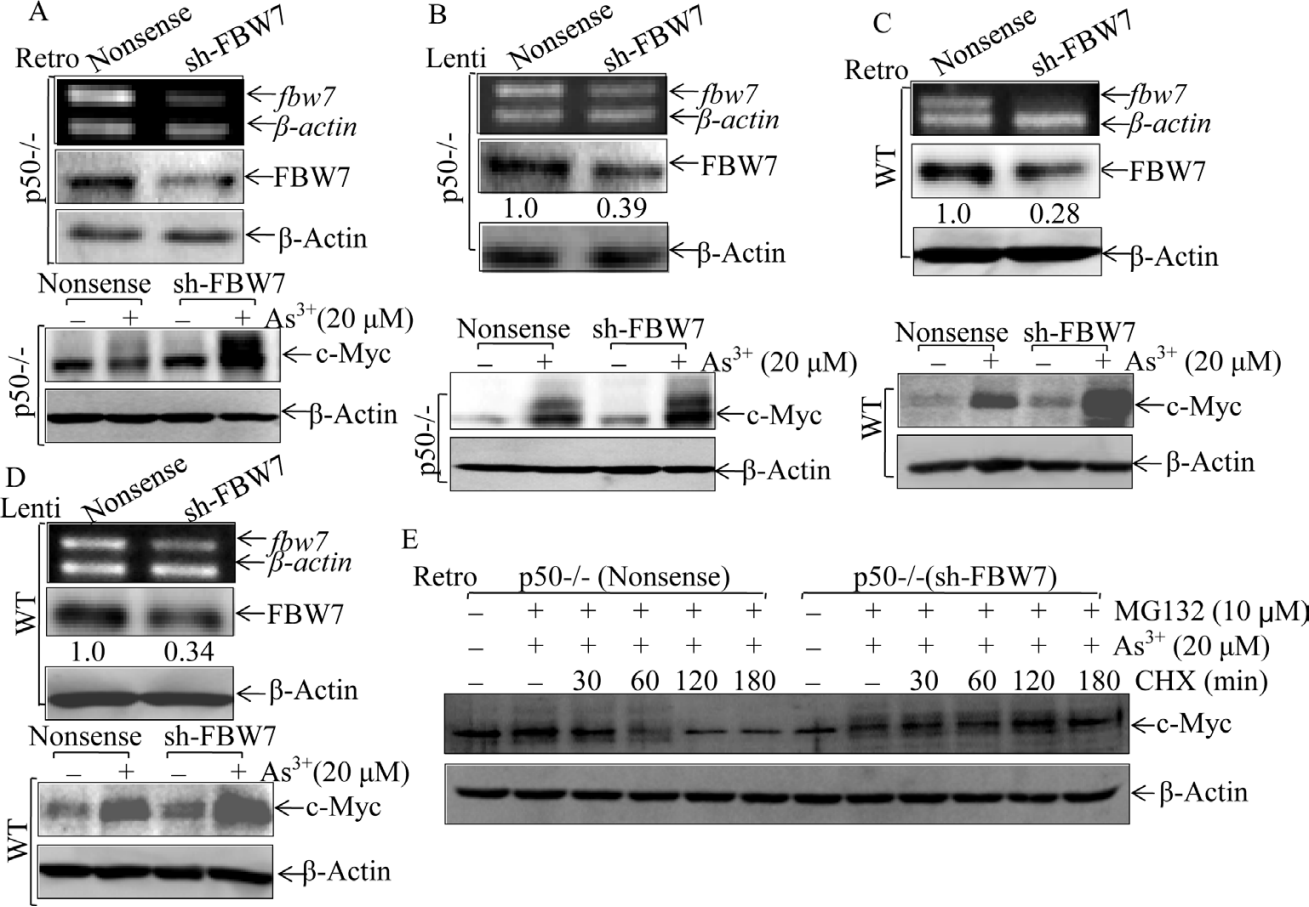
FBW7 was a p50 downstream mediator regulating c-Myc protein accumulation following arsenite treatment (A-D), Retroviral or Lentiviral constructs carrying mouse FBW7 shRNA were stably transfected in p50-/- (A & B) and WT (C & D) cells, and the efficiency of shRNA were determined by RT-PCR and Western Blotting. All of the transfectants were treated with 20 uM arsenite for 24 h, and cell extracts were subjected to Western Blotting. (E), c-Myc protein degradation rates were compared between p50-/-(nonsense) and p50-/-(sh-FBW7) cells

To provide a definite evidence regarding role of FBW7 in p50-mediated c-Myc protein accumulation upon arsenite treatment, FBW7 expression in p50-/- cells was stably knocked down using two pairs of shRNA (sh-FBW7). As shown in Fig. 5A, stable introduction of Retroviral-sh-FBW7 dramatically reduced FBW7 expression in p50-/- cells. Importantly, knockdown of FBW7 expression in p50-/- cells restored c-Myc protein expression upon arsenite treatment (Fig. 5A), demonstrating that FBW7 upregulation in p50-/- was responsible for defect of c-Myc induction by arsenite. The role of FBW7 in regulation of c-Myc protein expression was further extended in from using another pair of Lentiviral shRNA targeting mouse FBW7 and in WT cells with transfection of either Lentiviral FBW7 shRNA or Retroviral-FBW7 shRNA (Figs. 5B–5D). Moreover, we also determined whether the FBW7-downregulated c-Myc protein expression occurred at protein degradation, degradation assay has been performed. As shown in Fig. 5E, co-incubation with MG132 and arsenite led to accumulation of c-Myc protein in both p50-/-(sh-FBW7) and p50-/-(nonsense) cells. The marked protein degradation of accumulated c-Myc could be observed within 60 min in p50 (nonsense) upon CHX incubation, while this degradation was not seen for as long as 120 min in p50-/-(sh-FBW7) transfectants (Fig. 5E). These results depicted a critical role and mechanism of FBW7 upregulation for the defect of c-Myc protein expression in p50-/- cells following arsenite treatment.

### p50 suppresses FBW7 transcription through inhibiting E2F1 activation

**Fig 6:**
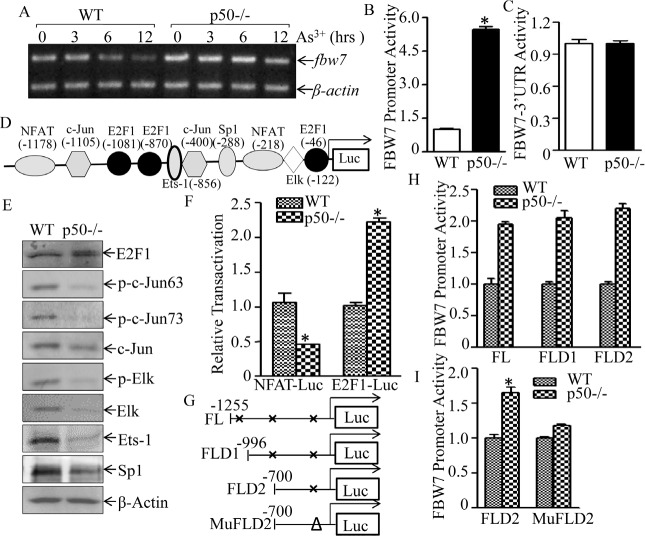
p50 suppressed fbw7 mRNA transcription by inhibiting E2F1 activation (A), WT and p50-/- cells were exposed to 20 μM arsenite and the mRNA level of FBW7 was determined by RT-PCR; fbw7 promoter-driven luciferase transcriptional activity (B) and fbw7−3′UTR(C) activity were analyzed and compared between WT and p50-/- cells. The results were shown as relative luciferase activity by normalized to TK and pGL3, respectively; (D), potential transcriptional factor binding sites in fbw7 promoter (−1255---+44, FL) were analyzed by TRANSFAC 8.3 engine online; (E), The cell extracts from WT and p50-/- cells were subjected to Western blotting for determination of c-Jun, Elk, Ets-1 and Sp1 protein expression and their phosphorylated status. (F), NFAT and E2F1 transactivation were determined and compared between WT and p50-/- cells by transient transfection of each of transcription factor-dependent luciferase reporters. the results were shown as relative luciferase activity by normalized to TK. (G), three potential E2F1 binding site deletion plasmids as indicated were constructed from full length of fbw7-promoter driven luciferase reporter. (H,I), relative activity of fbw7 promoter between WT and p50-/- was measured, and the results were presented as luciferase activity in p50-/- cells relative to WT cells with normalization to TK.

Although c-Myc has been reported to be a FBW7 target [35], the potential role of p50 in regulation of FBW7 and c-Myc degradation had never been explored. To evaluate the molecular mechanism underlying p50 suppression of FBW7 expression, fbw7 mRNA and transcription levels were detected in WT and p50-/- cells. Consistent with protein expression, both fbw7 mRNA and fbw7 promoter transcription activity were upregulated in p50-/- cells as comparison to these in WT cells (Fig. 6A & 6B). In contrast, fbw7 3'UTR-luciferase reporter activities were comparable between WT and p50-/- cells (Fig. 6C), conclusively demonstrated that p50-regulated FB W7 protein expression occurred at transcription level. With theoretical feasibility, we analyzed transcription factor binding sites within fbw7 promoter sequence using TRANSFAC 8.3 in PROMO HOME PAGE database, the potential transcription factor binding sites in fbw7 promoter were shown in Fig. 6D. The results obtained from comparison of expression and/or activation of those transcription factors between WT and p50-/-cells indicated that deletion of p50 upregulated E2F1 expression and transactivation, whereas it downregulated expression and/or activation of other transcription factors including c-Jun, Elk, Ets, Sp1 and NFAT (Figs. 6E and 6F), strongly suggesting that E2F1 might play role in p50-downregulated FBW7 expression. To elucidate a role of E2F1 in fbw7 promoter-driven transcriptional activity, two fragment deletions of E2F1 binding site (FLD1 and FLD2) and one potential binding sites mutant (MuFLD2) in fbw7 promoter-driven luciferase reporters shown in Fig. 6G were transiently transfected into both WT and p50-/- cells. The transfectants of FLD 1 and FLD2 showed a similar fbw7 promoter transcriptional activity, whereas 4 bp deletion of E2F1 binding site at -46 in FLD2 fbw7 promoter-driven luciferase reporter (MuFLD2) dramatically impaired the promoter transcriptional activity in comparison to that in FLD2 (Figs. 6H–6I), conclusively demonstrating that p50 downregulated fbw7 transcription in an E2F1-dependent manner.

### p50/FBW7/c-Myc contributes to arsenite-induced cell apoptosis

**Fig 7:**
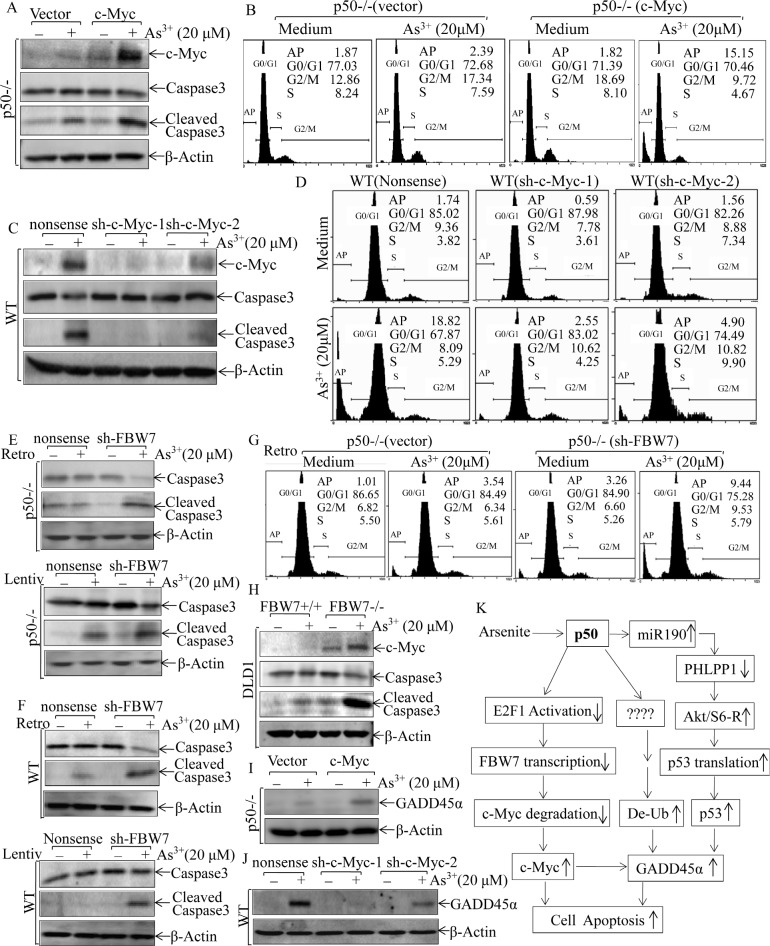
c-Myc induction mediated by FBW7 downregulation was required for apoptotic response following arsenite exposure (A, C, E, F, H, I & J), The stable transfectants as indicated were exposed to 20 μM arsenite for 24 h. Cell extracts were subjected to Western Blotting. (B, D & G) The indicated transfectants were exposed to 20 μM arsenite for 24 h, and the cells were then collected for cell flow cytometry analysis. (K) The diagram of p50 inhibition of c-Myc protein degradation and mediation of apoptotic response following arsenite exposure.

Our recent studies demonstrate that p50 is crucial for cell apoptotic response upon arsenite treatment [9, 10]. To test whether p50-mediated c-Myc accumulation contributed to arsenite-induced cell apoptosis, we stably transfected exogenous c-Myc expression construct into p50-/- cells. As shown in Fig. 7A, exogenous expression of c-Myc in p50-/- cells rendered p50-/- cells more sensitive to apoptotic response as compared with that in p50-/-(vector) cells, suggesting that restricted c-Myc protein accumulation in p50-/- cells mediated p50-/- cell resistance to apoptotic response upon arsenite treatment (Figs. 7A–B). The crucial role of c-Myc protein induction by arsenite treatment was consistently extended by the results obtained from knockdown of c-Myc expression in WT cells (Figs. 7C and 7D). Furthermore, the reduction of FBW7 expression by shRNA targeting FBW7 could restore the apoptotic responses following arsenite treatment in p50-/- cells (Figs. 7E–G). This observation was further strengthened by the consistent results obtained from using FBW7 knockout colon adenocarcinoma cell DLD1(FBW7-/-) (Fig. 7H). Thus, our results demonstrated that the regulation of FBW7/c-Myc was a novel mechanism underlying p50-mediated cell apoptotic response following arsenite treatment. To assess whether there is a cross-talk between p50/c-Myc pathway and p50/GADD45a cascade that is responsible for arsenite apoptotic response demonstrated in our previous studies [9], we evaluated the effect of c-Myc induction on GADD45α protein expression following arsenite treatment in either p50-/-(c-Myc) and WT (sh-c-Myc) transfectants. The results profoundly indicated that ectopic expression of c-Myc in p50-/- cells resulted in GADD45a protein upregulation (Fig. 7I), while knockdown of c-Myc by its shRNA in WT reduced GADD45a protein expression (Fig. 7J), demonstrating that p50-mediated c-Myc protein accumulation was an important positive factor for GADD45α protein upregulation following arsenite treatment.

## DISCUSSION

Our new findings regarding to p50 regulation of c-Myc protein degradation, coupled together with our previous reports on p50 in regulation of protein translation and modification, released the nature of p50 biological effects on the regulation of protein expression at multiple levels [9, 10] as schemed in Fig. 7K. Our published studies demonstrate that arsenite is associated with the promotion of cell cycle progression, cell proliferation and transformation via the NFκB transcription-dependent pathway [36, 37], while arsenite treatment leads to cell apoptosis through the p50-dependent and NFκB transcription-independent pathway [9]. Thus, p50-mediated NFκB transcription-dependent and -independent pathways may play a pivotal “switch-like” role for the determination of cell death and survival, explaining the bifurcated biological effects of arsenite treatment on carcinogenesis and cancer therapy. Therefore, understanding the molecular mechanisms underlying the arsenite-p50-mediated cell apoptotic pathway will provide key information for potential utilization of arsenite-p50 and its downstream components as targets for cancer chemoprevention and therapy.

Although arsenite-induced c-Myc expression was presumably regulated at transcriptional level [17], our current study exhibited that p50 regulates c-Myc expression via NF-κB transcription-independent cascade due to arsenite treatment. With more interesting we unexpectedly demonstrated that c-Myc was induced by arsenite at protein degradation level and p50 was essential for it. Since c-Myc has been reported to be involved in both cell apoptosis and cell transformation, our results will provide us important insight into understanding arsenite's carcinogenic and anti-cancer effects, which might be due to either the threshold value for c-Myc induction and levels or the c-Myc crosstalk with the induction of other key proteins, such as cyclin D1, COX-2 and GADD45α that have been documented in our published studies [36, 38].

c-Myc, as a stress response transcription factor, its degradation was precisely controlled by the complicated system. c-Myc protein could be phosphorylated by ERK1/2 at Ser62, which allows GSK3 to phosphorylate c-Myc at Thr58 [39], while phosphatase PP2A could de-phosphorylate c-Myc at Ser62 [29]. The phosphorylated Thr58 and dephosphorylated Ser62 could serve as a dock to recruit a Thr58 phosphorylation-dependent E3 ubiquitin ligase complex, called SCF^Fbw7^, and leading to c-Myc protein degradation [35]. Our results from current study excluded the possibility of ERK1/2 activation and PP2A or GSK3 expression involved in p50 regulation of c-Myc protein accumulation following arsenite treatment. Very interestingly, we found that FBW7 was upregulated in p50-/- cells and C141 p50 knockdown cells in comparison to that observed in their parental cells, suggesting that FBW7 might be a mediator for p50-regulated c-Myc protein accumulation due to arsenite treatment. Subsequently studies clearly demonstrated that FBW7 expression is inhibited by p50 and this inhibition is crucial for p50-mediated c-Myc protein accumulation due to arsenite treatment.

Although much is known about FBW7 which as an E3 ligase targeted proteins, including c-Myc, cyclin E, Notch, c-Jun, mTOR, MCL1 [40], relatively little is known about mechanisms underlying modulating FBW7 expression. C/EBPδ has been shown to bind to fbw7 gene promoter and inhibit its transcription [41]. whereas miR-223 and miR-27a can downregulate FBW7 expression due to targeting the fbw7 3′-untranslated region [42, 43]. In this study, we discovered a novel mechanism underlying FBW7 regulation that p50 decreased fbw7 mRNA level by inhibiting E2F1-mediated fbw7 promoter transcriptional activation. FBW7 is a multiple-function protein that acts as a tumor suppressor and plays multiple roles in the regulation of cell division and differentiation [20, 44]. Recent publications interestingly highlight the function of FBW7 on cell apoptosis [44–46]. The depletion of fbw7 in the mouse brain caused severely impaired stem cell differentiation and increased progenitor cell death via Notch and c-Jun. FBW7-deficient human T-ALL cell lines were more sensitive to sorafenib (a drug approved for the treatment of primary kidney cancer) via regulation of MCL1 degradation. These studies suggest that FBW7 regulates cell apoptotic responses through differential mechanisms. Our present study found that p50-mediated c-Myc upregulation by inhibiting FBW7 expression contributed to arsenite-induced cell apoptosis. Collectively, our results were not only depicted a novel mechanism regarding the regulation of FBW7 expression, but also provided new insight of p50's biological function, expanding the function of p50 across multiple new fields of research.

c-Myc induction by arsenite has been reported that linked to arsenite effects on cell transformation and tumorigenesis [17]. In our present study, however, we highlight the function of c-Myc in p50-mediated cell apoptosis following arsenite treatment. Knockdown of c-Myc in WT cells led to a resistant to arsenite-induced apoptosis, while over-expression c-Myc in p50-/- cells restored the apoptotic sensitivity to arsenite. These results strongly suggested that c-Myc was in fact required for arsenite-induced apoptosis. Our previous study identified that GADD45α played an essential role in p50-mediated cell death [9]. The results from the present study showed that knockdown of c-Myc in WT cells decreased the arsenite-induced GADD45α expression, while overexpression of c-Myc in p50-/- cells promoted GADD45α induction, strongly suggesting a positive link to connect p50/c-Myc to p50/GADD45α apoptotic cascades. Although c-Myc has been reported to contribute to the repression of GADD45α expression [47], we noted in our study that ectopic expression of c-Myc only in p50-/- cells failed to induce basal GADD45α expression and apoptosis, while arsenite-induced apoptosis in p50-/-(c-Myc) cells has been elevated as compared with that in p50-/-(vector) transfectant. This result is consistent with previous finding that fibroblasts with ectopic c-Myc expression undergo to apoptosis in cell response to environmental stresses, such as hypoxia [48]. Previous studies primarily focused on c-Myc expression in the carcinogen effects of arsenite, whereas our current study for the first time link c-Myc induction to arsenite-induced cell apoptosis. Consideration of the induction of cell apoptosis of p50/FBW7/c-Myc pathway consistent with potential anticancer strategies, our results may lead to new targets for chemoprevention of cancer and providing new basic knowledge of arsenite as a chemical medicine.

## METHODS

### Reagents and Plasmids

Arsenite (As^3+^) was purchased from Aldrich (Milwaukee, WI, USA). Proteasome chemical inhibitor MG132, MEK1/2 inhibitor PD98059 and protein synthesis inhibitor cyclohexamide (CHX) were bought from Calbiochem (San Diego, CA, USA). The dual luciferase assay kit, TRIzol reagent and SuperScript^™^ First-Strand Synthesis system were obtained from Promega (Madison, WI, USA) and Invitrogen (Grand Island, NY, USA), respectively. Poly Jet^™^ DNA In Vitro Transfection Reagent was purchased from SignaGen Laboratories (Rockville, MD, USA). λ-PPase kit was purchased from New England Biolabs (Ipswich, MA, USA). The plasmids of shRNA p50 (sh-p50), shRNA CSN2 (sh-CSN2), shRNA CSN5 (sh-CSN5) and its control vector were purchased from Open Biosystems (Thermo Fisher Scientific, Pittsburgh, PA, USA). The fbw7 promoter(-1255-+44)-driven luciferase reporter plasmid [41] and fbw7−3′ UTR-luciferase reporter construct [42] were gifts from Dr. Esta Sterneck (National Cancer Institute, Frederick, MD, USA) and Dr. Alex C. Minella (Northwestern University Feinberg School of Medicine, Chicago, IL, USA), respectively. Lentivirus and retrovirus plasmid specific targeting mouse fbw7 (sh-Fbw7) [49] were kindly provided by Dr. Iannis Aifantis (New York University School of Medicine, New York, NY, USA). c-Myc expression construct [29] was from Dr. Rosalie Sears (Oregon Health & Science University, Portland, OR, USA).

### Cell culture and Transfection

The p50-/- and p65-/- MEFs and their corresponding wild type (WT) MEFs were cultured as described in our previous studies [9]. NIH3T3 cells were maintained in DMEM (Invitrogen, Carlsbad, CA, USA) supplemented with 10% FBS (FBS, Nova-Tech, Grand Island, NE, USA), 1% penicillin/streptomycin, and 2 mM L-glutamine (Life Technologies, Grand Island, NY, USA) at 37°C. Mouse epidermal JB6 Cl41 cells were cultured in MEM with 5% FBS. FBW7-/- and its parental DLD1 cells [50] were kind gifts from Dr. Bert Vogelstein (The Johns Hopkins University, Baltimore, MD, USA), and cultured in 10% FBS/Micro5A medium (Invitrogen, Carlsbad, CA, USA).

The stable cell lines of p50-/-(p50) and p65-/-(p65) were established and described in our previous publications [9]. Cell transfections were performed with PolyJet^™^ DNA In Vitro Transfection Reagent, according to the manufacturer's protocol. For stable transfection, cultures were subjected to either puromycin (Alexis, Plymouth, PA, USA) or G418 (Invitrogen, Carlsbad, CA, USA) drug selection. The surviving cells that from the drug selection was pooled as stable mass culture. For transient transfection, cells were seeded and cultured in each well of 6-well plates for 24 h, and the cells were then transfected with either fbw7 promoter-luciferase reporter for determination of fbw7 promoter transcriptional activity or indicated transcription factor-luciferase reporter construct for determination of transactivation of related transcription factor.

### RT-PCR

Cells were treated with arsenite for the time points as indicated, and the cells were extracted and then 5μg total RNA was used for first-strand cDNA synthesis with oligdT (20) primer by SuperScript^™^ First-Strand Synthesis system (Invitrogen). Two pairs of oligonucleotides (Forward: 5′-TCCTGTACCTCGTCCGATTC-3′, Reverse: 5′-AATTCAGGGATCTGGTCAC G-3′; and Forward: 5′-CCTAAAGAGTTGGCACTCTATG-3′ Reverse: 5′-ACTCCACCTGT ATGTCCCACT-3′) were used as the specific primers to amplify mouse c-myc and mouse fbw7, respectively. β-actin was used as its loading control [10]. The results were imagined with Alpha Innotech SP image system (Alpha Innotech Corporation, San Jose, CA, USA).

### Flow Cytometry

Cells (3 × 10^5^) were seeded into each well of 6-well Plates, and cultured up to ~80% confluence. Cell culture medium will be replaced with 0.1% FBS DMEM medium for 24 h. The cells were then exposed to arsenite for 24 h, and the cells were then collected for Propidium Iodide (PI) staining and then subjected to Flow Cytometry analysis [51].

### Antibodies and Western Blot

The antibodies specific against p-GSK-3α/β, GSK-3β, p-PP2A, PP2A, p-ERK1/2, ERK1/2, Skp1, Skp2, CSN2, CSN5, p-AKT473, Caspase-3 were purchased from Cell Signaling Technology (Beverly, MA, USA). Antibodies specific against c-Myc, p-c-Myc at T58/S62 and Cul4A were bought from Santa Cruz Biotechnology (Santa Cruz, CA, USA). Antibodies specific against USP28 and p50 were bought from Abcam (Cambridge, MA, USA). Fbw-7 antibody and HA antibody were obtained from Aviva Systems Biology (San Diego, CA, USA) and Covance Inc. (Princeton, NJ, USA),respectively. Antibodies against β-Actin and α-Tubulin were bought from Sigma (St. Louis, MO, USA). Western blotting was performed as described in our previous publication [52].

### De-phosphorylation Assay

Arsenite-treated cells were collected and re-suspended in PBS. The cells were then homogenized by sonication, and the whole cell lysate (100 μg in 40μl) was used as substrate and incubated with λ phosphatase in the phosphatase buffer supplied by the manufacturer (New England Biolabs, Ipswich, MA, USA) for 1 h at 37 °C. Phosphorylation of the protein was evaluated by Western Blotting [53].

### Luciferase Reporter Assay

fbw7 promoter-luciferase, fbw7 3′ UTR-luciferase reporter, E2F1 or NFAT-dependent luciferase reporter plasmids were transiently co-transfected with pRL-TK into cells of 96-well plates (8×10^3^ per well) and subjected to the various indicated treatments. To detect the E2F1 binding sites, we constructed two short fbw7 promoter (FLD1 and FLD2) at -996 or -700 position using following primers: (F1):5′-GGGGTACCCATCCGAGAGATCC AGTCC-3(F2): 5′-GGGGTACCCAGAGCTTCTGCCTCGT

CC-3′, (R1): 5′-CCCAAGCTTGGGTGGTTCCCT TCCTCCTTCGGACTG-3′. We also constructed 4bp predicted E2F1 binding sites deletion at -46 position on fbw7 promoter (MuFLD2) using a pair of primer: (F3):5′-CGGAAGAGACCCGCTGGTTTAGCGACAC-3′, (R2): 5′-GTGTCGCTAAACCAG CCGGG TCTCTTCCG-3′. Luciferase activities were determined as described previously [54].

### Statistical Methods

Student's t test was employed to determine the significance of differences between various groups. The differences will be considered significant at p<0.05.

## References

[1] Lawrence T (2009). The nuclear factor NF-kappaB pathway in inflammation. Cold Spring Harb Perspect Biol.

[2] Sethi G, Ahn KS, Aggarwal BB (2008). Targeting nuclear factor-kappa B activation pathway by thymoquinone: role in suppression of antiapoptotic gene products and enhancement of apoptosis. Mol Cancer Res.

[3] Wang S, Liu Z, Wang L, Zhang X (2009). NF-kappaB signaling pathway, inflammation and colorectal cancer. Cell Mol Immunol.

[4] Dale E, Davis M, Faustman DL (2006). A role for transcription factor NF-kappaB in autoimmunity: possible interactions of genes, sex, and the immune response. Adv Physiol Educ.

[5] Kaltschmidt B, Kaltschmidt C (2009). NF-kappaB in the nervous system. Cold Spring Harb Perspect Biol.

[6] Hayden MS, Ghosh S (2004). Signaling to NF-kappaB. Genes Dev.

[7] Hayden MS, Ghosh S (2012). NF-kappaB, the first quarter-century: remarkable progress and outstanding questions. Genes Dev.

[8] Cao S, Zhang X, Edwards JP, Mosser DM (2006). NF-kappaB1 (p50) homodimers differentially regulate pro- and antiinflammatory cytokines in macrophages. J Biol Chem.

[9] Song L, Li J, Zhang D, Liu ZG, Ye J, Zhan Q, Shen HM, Whiteman M, Huang C (2006). IKKbeta programs to turn on the GADD45alpha-MKK4-JNK apoptotic cascade specifically via p50 NF-kappaB in arsenite response. J Cell Biol.

[10] Yu Y, Zhang D, Huang H, Li J, Zhang M, Wan Y, Gao J, Huang C (2013). NF-kappaB1 p50 promotes p53 protein translation through miR-190 downregulation of PHLPP1. Oncogene.

[11] Dong Z (2002). The molecular mechanisms of arsenic-induced cell transformation and apoptosis. Environ Health Perspect.

[12] Zhang XW, Yan XJ, Zhou ZR, Yang FF, Wu ZY, Sun HB, Liang WX, Song AX, Lallemand-Breitenbach V, Jeanne M, Zhang QY, Yang HY, Huang QH, Zhou GB, Tong JH, Zhang Y (2010). Arsenic trioxide controls the fate of the PML-RARalpha oncoprotein by directly binding PML. Science.

[13] Ouyang W, Luo W, Zhang D, Jian J, Ma Q, Li J, Shi X, Chen J, Gao J, Huang C (2008). PI-3K/Akt pathway-dependent cyclin D1 expression is responsible for arsenite-induced human keratinocyte transformation. Environ Health Perspect.

[14] Lu TH, Su CC, Chen YW, Yang CY, Wu CC, Hung DZ, Chen CH, Cheng PW, Liu SH, Huang CF (2011). Arsenic induces pancreatic beta-cell apoptosis via the oxidative stress-regulated mitochondria-dependent and endoplasmic reticulum stress-triggered signaling pathways. Toxicol Lett.

[15] Lau AT, Li M, Xie R, He QY, Chiu JF (2004). Opposed arsenite-induced signaling pathways promote cell proliferation or apoptosis in cultured lung cells. Carcinogenesis.

[16] Trouba KJ, Geisenhoffer KM, Germolec DR (2002). Sodium arsenite-induced stress-related gene expression in normal human epidermal, HaCaT, and HEL30 keratinocytes. Environ Health Perspect.

[17] Chen H, Liu J, Zhao CQ, Diwan BA, Merrick BA, Waalkes MP (2001). Association of c-myc overexpression and hyperproliferation with arsenite-induced malignant transformation. Toxicol Appl Pharmacol.

[18] Boone DN, Qi Y, Li Z, Hann SR (2011). Egrl mediates p53-independent c-Myc-induced apoptosis via a noncanonical ARF-dependent transcriptional mechanism. Proc Natl Acad Sci USA.

[19] Sakamuro D, Eviner V, Elliott KJ, Showe L, White E, Prendergast GC (1995). c-Myc induces apoptosis in epithelial cells by both p53-dependent and p53-independent mechanisms. Oncogene.

[20] Wang Z, Inuzuka H, Fukushima H, Wan L, Gao D, Shaik S, Sarkar FH, Wei W (2011). Emerging roles of the FBW7 tumour suppressor in stem cell differentiation. EMBO Rep.

[21] Gomez-Caminero A, Howe P, Hughes M, Kenyon E, Lewis DR, Moore M, Ng J, Aitio A, Becking G (2001). Arsenic and Arsenic Compounds. World Health Organization, Geneva.

[22] Nakadaira H, Endoh K, Katagiri M, Yamamoto M (2002). Elevated mortality from lung cancer associated with arsenic exposure for a limited duration. Journal of Occupational and Environmental Medicine.

[23] Pi J, Yamauchi H, Kumagai Y, Sun G, Yoshida T, Aikawa H, Hopenhayn-Rich C, Shimojo N (2002). Evidence for induction of oxidative stress caused by chronic exposure of Chinese residents to arsenic contained in drinking water. Environmental Health Perspectives.

[24] Smith AH, Goycolea M, Haque R, Biggs ML (1998). Marked Increase in Bladder and Lung Cancer Mortality in a Region of Northern Chile Due to Arsenic in Drinking Water. Am J Epidemiol.

[25] Che X, Liu J, Huang H, Mi X, Xia Q, Li J, Zhang D, Ke Q, Gao J, Huang C (2013). p27 suppresses cyclooxygenase-2 expression by inhibiting p38beta and p38delta-mediated CREB phosphorylation upon arsenite exposure. Biochim Biophys Acta.

[26] Shimizu M, Hochadel IF, Fulmer BA, Waalkes MP (1998). Effect of glutathione depletion and metallothionein gene expression on arsenic-induced cytotoxicity and c-myc expression in vitro. Toxicol Sci.

[27] La Rosa FA, Pierce JW, Sonenshein GE (1994). Differential regulation of the c-myc oncogene promoter by the NF-kappa B rel family of transcription factors. Mol Cell Biol.

[28] Palomero T, Lim WK, Odom DT, Sulis ML, Real PJ, Margolin A, Barnes KC, O'Neil J, Neuberg D, Weng AP, Aster JC, Sigaux F, Soulier J, Look AT, Young RA, Califano A (2006). NOTCH1 directly regulates c-MYC and activates a feed-forward-loop transcriptional network promoting leukemic cell growth. Proc Natl Acad Sci USA.

[29] Arnold HK, Sears RC (2006). Protein phosphatase 2A regulatory subunit B56alpha associates with c-myc and negatively regulates c-myc accumulation. Mol Cell Biol.

[30] Dai MS, Jin Y, Gallegos JR, Lu H (2006). Balance of Yin and Yang: ubiquitylation-mediated regulation of p53 and c-Myc. Neoplasia.

[31] Gregory MA, Qi Y, Hann SR (2003). Phosphorylation by glycogen synthase kinase-3 controls c-myc proteolysis and subnuclear localization. J Biol Chem.

[32] Glickman MH, Ciechanover A (2002). The ubiquitin-proteasome proteolytic pathway: destruction for the sake of construction. Physiol Rev.

[33] Richardson KS, Zundel W (2005). The emerging role of the COP9 signalosome in cancer. Mol Cancer Res.

[34] Dominguez-Sola D, Dalla-Favera R (2004). PINning down the c-Myc oncoprotein. Nat Cell Biol.

[35] Welcker M, Orian A, Jin J, Grim JE, Harper JW, Eisenman RN, Clurman BE (2004). The Fbw7 tumor suppressor regulates glycogen synthase kinase 3 phosphorylation-dependent c-Myc protein degradation. Proc Natl Acad Sci U S A.

[36] Ouyang W, Ma Q, Li J, Zhang D, Liu Z-g, Rustgi AK, Huang C (2005). Cyclin D1 Induction through I {kappa} B Kinase {beta}/Nuclear Factor-{kappa}B Pathway Is Responsible for Arsenite-Induced Increased Cell Cycle G1-S Phase Transition in Human Keratinocytes. Cancer Res.

[37] Ouyang W, Li J, Ma Q, Huang C (2006). Essential roles of PI-3K/Akt/IKK{beta}/NF{kappa}B pathway in cyclin D1 induction by arsenite in JB6 C141 cells. Carcinogenesis.

[38] Ouyang W, Zhang D, Ma Q, Li J, Huang C (2007). Cyclooxygenase-2 induction by arsenite through the IKKbeta/NFkappaB pathway exerts an antiapoptotic effect in mouse epidermal C141 cells. Environ Health Perspect.

[39] Sears R, Nuckolls F, Haura E, Taya Y, Tamai K, Nevins JR (2000). Multiple Ras-dependent phosphorylation pathways regulate Myc protein stability. Genes Dev.

[40] Welcker M, Clurman BE (2007). Fbw7/hCDC4 dimerization regulates its substrate interactions. Cell Div.

[41] Balamurugan K, Wang JM, Tsai HH, Sharan S, Anver M, Leighty R, Sterneck E (2010). The tumour suppressor C/EBPdelta inhibits FBXW7 expression and promotes mammary tumour metastasis. EMBO J.

[42] Xu Y, Sengupta T, Kukreja L, Minella AC (2010). MicroRNA-223 regulates cyclin E activity by modulating expression of F-box and WD-40 domain protein 7. J Biol Chem.

[43] Lerner M, Lundgren J, Akhoondi S, Jahn A, Ng HF, Akbari Moqadam F, Oude Vrielink JA, Agami R, Den Boer ML, Grander D, Sangfelt O (2011). MiRNA-27a controls FBW7/hCDC4-dependent cyclin E degradation and cell cycle progression. Cell Cycle.

[44] Hoeck JD, Jandke A, Blake SM, Nye E, Spencer-Dene B, Brandner S, Behrens A (2010). Fbw7 controls neural stem cell differentiation and progenitor apoptosis via Notch and c-Jun. Nat Neurosci.

[45] Nateri AS, Riera-Sans L, Da Costa C, Behrens A (2004). The ubiquitin ligase SCFFbw7 antagonizes apoptotic JNK signaling. Science.

[46] Inuzuka H, Shaik S, Onoyama I, Gao D, Tseng A, Maser RS, Zhai B, Wan L, Gutierrez A, Lau AW, Xiao Y, Christie AL, Aster J, Settleman J, Gygi SP, Kung AL (2011). SCF(FBW7) regulates cellular apoptosis by targeting MCL1 for ubiquitylation and destruction. Nature.

[47] Barsyte-Lovejoy D, Mao DY, Penn LZ (2004). c-Myc represses the proximal promoters of GADD45a and GADD153 by a post-RNA polymerase II recruitment mechanism. Oncogene.

[48] Rupnow BA, Alarcon RM, Giaccia AJ, Knox SJ (1998). p53 mediates apoptosis induced by c-Myc activation in hypoxic or gamma irradiated fibroblasts. Cell Death Differ.

[49] Reavie L, Delia Gatta G, Crusio K, Aranda-Orgilles B, Buckley SM, Thompson B, Lee E, Gao J, Bredemeyer AL, Helmink BA, Zavadil J, Sleckman BP, Palomero T, Ferrando A, Aifantis I (2010). Regulation of hematopoietic stem cell differentiation by a single ubiquitin ligase-substrate complex. Nat Immunol.

[50] Rajagopalan H, Jallepalli PV, Rago C, Velculescu VE, Kinzler KW, Vogelstein B, Lengauer C (2004). Inactivation of hCDC4 can cause chromosomal instability. Nature.

[51] Fang Y, Yu Y, Hou Q, Zheng X, Zhang M, Zhang D, Li J, Wu XR, Huang C (2012). The Chinese herb isolate isorhapontigenin induces apoptosis in human cancer cells by down-regulating overexpression of antiapoptotic protein XIAP. J Biol Chem.

[52] Yu Y, Li J, Wan Y, Lu J, Gao J, Huang C (2013). GADD45alpha Induction by Nickel Negatively Regulates JNKs/p38 Activation via Promoting PP2Calpha Expression. PLoS One.

[53] Zhang D, Li J, Zhang M, Gao G, Zuo Z, Yu Y, Zhu L, Gao J, Huang C (2012). The requirement of c-Jun N-terminal kinase 2 in regulation of hypoxia-inducing factor-1alpha mRNA stability. J Biol Chem.

[54] Zuo Z, Cai T, Li J, Zhang D, Yu Y, Huang C (2012). Hexavalent chromium Cr(VI) up-regulates COX-2 expression through an NFkappaB/c-Jun/AP-1-dependent pathway. Environ Health Perspect.

